# Dissecting the Role of Subtypes of Gastrointestinal Vagal Afferents

**DOI:** 10.3389/fphys.2020.00643

**Published:** 2020-06-11

**Authors:** Yoko B. Wang, Guillaume de Lartigue, Amanda J. Page

**Affiliations:** ^1^Vagal Afferent Research Group, Adelaide Medical School, The University of Adelaide, Adelaide, SA, Australia; ^2^Department of Pharmacodynamics, College of Pharmacy, University of Florida, Gainesville, FL, United States; ^3^Center for Integrative Cardiovascular and Metabolic Disease, University of Florida, Gainesville, FL, United States; ^4^Nutrition, Diabetes and Gut Health, Lifelong Health Theme, South Australian Health and Medical Research Institute, Adelaide, SA, Australia

**Keywords:** gastrointestinal tract, vagal afferent subtypes, gut brain axis, molecular tools, feeding behaviour

## Abstract

Gastrointestinal (GI) vagal afferents convey sensory signals from the GI tract to the brain. Numerous subtypes of GI vagal afferent have been identified but their individual roles in gut function and feeding regulation are unclear. In the past decade, technical approaches to selectively target vagal afferent subtypes and to assess their function has significantly progressed. This review examines the classification of GI vagal afferent subtypes and discusses the current available techniques to study vagal afferents. Investigating the distribution of GI vagal afferent subtypes and understanding how to access and modulate individual populations are essential to dissect their fundamental roles in the gut-brain axis.

## Introduction

The vagus nerve provides bidirectional communication between the gut and the brain. The vagus nerve comprises of both sensory and motor neurons with the number of afferent fibres out-numbering the efferent fibres by about 9 to 1 (Agostoni et al., 1957). Vagal sensory pathways facilitate signal transmission from the visceral endings in the gut through the vagal ganglia, where the cell bodies are located, and terminate in the brainstem. Visceral projection of vagal afferents is highly prevalent in the upper gastrointestinal (GI) tract and the density is gradually decreased further down the gut (Berthoud and Neuhuber, 2000). Instead, the lower GI tract is densely innervated by spinal afferents whose cell bodies lie in the dorsal root ganglia (DRG) (Spencer et al., 2016b). The GI vagal afferent cell bodies are located in the nodose ganglia (NG), originating from the epibranchial placode (Baker and Bronner-Fraser, 2001). The predominant function of this traffic is to transmit innocuous signals evoked by food related stimuli in the GI tract. In addition, a small number of jugular vagal afferents, with cell bodies in the jugular ganglia, also project to GI regions (Yu et al., 2005). The jugular neurons are derived from the neural crest, the same cell source as spinal afferent neurons. They share similarities in gene expression of somatosensory markers (Kupari et al., 2019), suggesting a possible similar function of jugular neurons and spinal afferents. Furthermore, vagal afferents also form direct monosynaptic connexions (Rinaman et al., 1989) and indirect interactions (via second order neurons) (Grabauskas and Owyang, 2017) with the efferent fibres in the nucleus tractus solitarius (NTS) to regulate the vago-vagal reflex. Vagal efferents, with the cell bodies located in the dorsal motor nucleus of the vagus (DMV), relay signals from the brain to the gut coordinating motor responses to maintain digestive function (Rogers et al., 1995).

GI vagal afferents play an important role in the regulation of food intake and GI function, orchestrating both physiological and behavioural aspects of food intake in order to ensure energy requirements are maintained. The peripheral afferent endings are specialised to detect mechanical and chemical stimuli evoked within the GI tract in response to food intake. These signals are transmitted to the brainstem and processed before being relayed to different regions of the brain, involved in physiological and behavioural function, or reflexing back to the GI tract to impact on gut motility and enzyme secretion (Travagli and Anselmi, 2016; Browning et al., 2017; Han et al., 2018; Suarez et al., 2018; Waise et al., 2018). It is known that there are numerous subtypes of GI vagal afferent depending on morphology and response to food related stimuli (Berthoud and Powley, 1992; Page et al., 2002). However, their individual role in feeding regulation and GI function is unclear. Identification of the role of specific subtypes of GI vagal afferent will provide critical information for the targeted treatment of disease.

This review will examine the different classifications of GI vagal afferents and discuss the current advances in technology that can improve understanding of the specific role these subclasses of GI vagal afferent play in gut function and appetite regulation.

## The Curious Case of Vagal Afferent Subtypes

**FIGURE 1:**
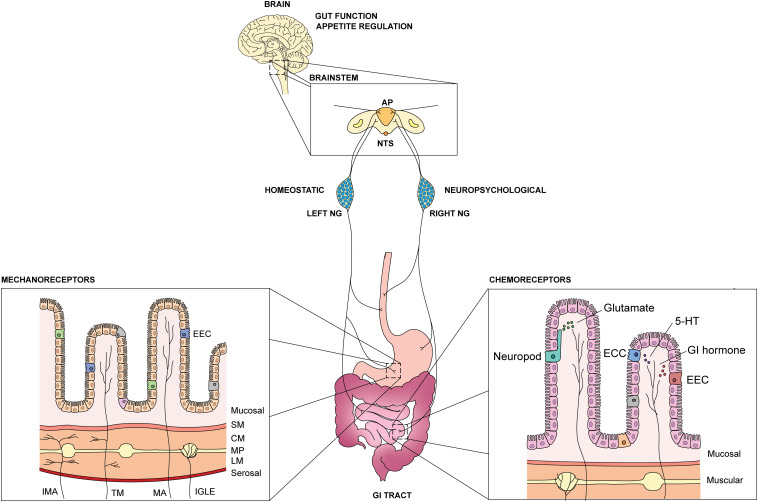
Overview of GI vagal afferents in the gut-brain axis. An illustration of GI vagal afferents neuroanatomy and primary sensory responses in the viscera. NTS: nucleus tractus solitarius, AP: area postrema, NG: nodose ganglia, EEC: enteroendocrine cells, ECC: enterochromaffin cells, 5-HT: 5-hydroxytriptamine, SM: submucosal, CM: circular muscle, MP: myenteric plexus, LM: longitudinal muscle, IMA: intramuscular arrays, TM: tension-mucosal afferents, MA: mucosal afferents, IGLE: intraganglionic laminar endings.

**FIGURE 2:**
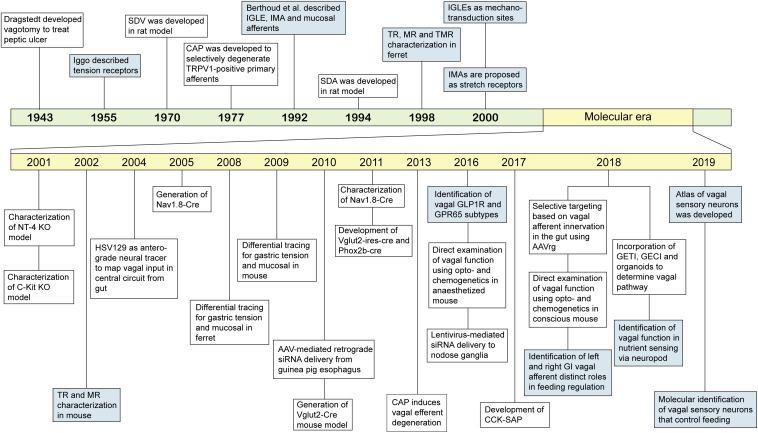
Landmark events of classification and technical advances to study GI vagal afferents. An overview of progress in understanding GI vagal afferents function and technical development to target vagal afferent subtypes. SDV: subdiaphragmatic vagotomy, CAP: capsaicin, TRPV1: transient receptor potential vanilloid 1, IGLE: intraganglionic laminar endings, IMA: intramuscular arrays, SDA: subdiaphragmatic deafferentation, TR: tension receptor, MR: mucosal receptor, TMR: tension-mucosal receptor, NT-4: neurotrophin-4, HSV129: herpes simplex virus strain 129, AAV: adeno-associated virus, GLP1R: glucagon like peptide 1 receptor, GPR65: G-coupled protein receptor 65, siRNA: small interfering RNA, CCK-SAP: cholecystokinin-saporin, AAVrg: adeno-associated virus retrograde, GETI: genetically encoded transmitter indicator, GECI: genetically encoded calcium indicator.

GI vagal afferent subtypes have been previously classified based on their characteristics, such as the embryonic origins, conductance velocity, anatomical and morphological organisation, response to primary stimuli, as well as defined molecular markers (Figure 1 and Table 1). In recent years, gene expression profiles (Egerod et al., 2018; Kupari et al., 2019) and neural circuits (Williams et al., 2016; Han et al., 2018; Kaelberer et al., 2018) have been introduced as new ways to categorise vagal afferent subtypes (Figure 2). Although all of these approaches aim to associate GI vagal afferents to their physiological and behavioural function, each classification has limitations when it comes to *in vivo* studies.

**TABLE 1:** Summary of GI vagal afferents classification.

Characteristics			Oesophagus	Stomach	Small Intestine	References
**Embryonic origins**						
	Neural crest-derived		+	−	−	Yu et al., 2005; Surdenikova et al., 2012; Trancikova et al., 2018
	Placode-derived		+	+	+	
**Conductance velocity**			Aδ- and C-fibre	C-fibre	C-fibre	Page and Blackshaw, 1998; Yu et al., 2005
**Visceral endings**						
	Muscle layer					
		IGLE	+++	+++	++	
		IMA	LES	Pyloric sphincter	Scarce	Rodrigo et al., 1970, 1975a,b; Pedrosa et al., 1976; Berthoud and Powley, 1992; Fox et al., 2000; Powley, 2000; Powley and Phillips, 2011; Powley et al., 2011, 2012, 2013, 2014, 2016
	Mucosal afferents		+	+	+	
	Mucosal-muscle afferents		+	N/A	N/A	
**Brainstem endings**						
	Nucleus tractus solitarius		NTS centralis	NTS medialis, NTS gelatinosus	NTS commisuralis	Travagli and Anselmi, 2016
	Area postrema		N/A	+	+	Han et al., 2018
**Primary response to stimuli**						
	Mechanosensitive		Tension receptor, Mucosal receptor, Tension-mucosal receptor	Tension receptor, Mucosal receptor	Tension receptor	Page and Blackshaw, 1998; Page et al., 2002
	Chemosensitive		Osmolarity, pH	Osmolarity, pH	Nutrient	Powley and Phillips, 2004; Williams et al., 2016
	Thermosensitive		+	+	+	El Ouazzani and Mei, 1982; El Ouazzani, 1984; Lennerz et al., 2007; Jänig, 2018
**Genetic markers**						
	Mucosal afferents		N/A	Sst/Gpr65, Calca	Glpr1R, Vip/Uts2b, Gpr65	Bai et al., 2019
	IGLE		N/A	GLP1R	Oxtr	
	IMA		N/A	Calca	N/A	
**Physiological function**			Baroreceptors, Nociceptors	Mechanosensing	Nutrient sensing	Yu et al., 2005; Kollarik et al., 2010b; Williams et al., 2016; Han et al., 2018; Kaelberer et al., 2018

### Vagal Afferent Neuroanatomy

Neuroanatomically, vagal afferents are often classified based on their ganglion of origins and conductance velocity. The former differentiates vagal afferents into jugular and nodose neurons, achieved by injecting neural tracers or performing immunostaining. However, this approach appears to be difficult since there are no molecular markers identified to be selectively expressed in the cell bodies of either the nodose or jugular ganglia. Furthermore, anatomical structure in certain animal models, i.e., mouse, limits the accuracy of separation at the level of the vagal ganglia since it is difficult to anatomically distinguish nodose and jugular ganglia (Nassenstein et al., 2010; Surdenikova et al., 2012). In a recent study, Kupari et al. (2019) distinguished nodose and jugular neurons based on the expression of *Phox2b* and *Prdm12*, respectively. This study suggests that vagal ganglia consist of 85% of nodose neurons and 15% of jugular neurons, of which eighteen subtypes of nodose neuron and six subtypes of jugular neuron have been identified (Kupari et al., 2019). Although a role for each subtype has been proposed in this study, based on the key molecular markers expressed, the physiological function of these subtypes requires further confirmation *in vitro* and *in vivo*.

Defining vagal afferent subtypes based on their signal transmission speed classifies vagal afferent neurons (VAN) into myelinated, fast conducting A-fibres, moderately myelinated, medium conducting B-fibres and unmyelinated, slow conducting C-fibres. This classification follows Erlanger-Gasser rules of afferent and efferent fibres based on their electrophysiological characteristics (Erlanger and Gasser, 1930). In addition, a secondary approach evaluating microscopic cellular structures (e.g., cell shape and surface characteristic) has also been established to identify nodose A- and C-fibre neurons in culture (Lu et al., 2013). However, it seems to be impractical for *in vivo* usage. Vagal A-fibres convey afferent visceral information and motor input, vagal B-fibres carry parasympathetic input, while vagal C-fibres deliver afferent visceral information (Ruffoli et al., 2011). Vagal afferent A- and C-fibres project to the GI tract, although, the ratio of composition may vary depending on the region of the GI tract innervated. For instance, the oesophagus is innervated by A- and C-fibre to a similar degree while subdiaphragmatic GI organs are predominantly innervated by C-fibres (Yu et al., 2005; Grabauskas and Owyang, 2017). Intriguingly, conductance velocity does not appear to be directly related to vagal afferent function with the location of innervation possibly acting as the main determinant of vagal afferent function (Page et al., 2002; Yu et al., 2005). Furthermore, the threshold of activation of vagal afferent fibres has also been associated with their physiological function. Low threshold activation is related to non-nociceptive function, such as in mechanosensitive tension and mucosal receptors (Page et al., 2002), whilst high threshold activation is linked to nociceptive-like characteristic, such as nodose C-fibres and jugular A-/C-fibres innervating the oesophagus (Yu et al., 2005).

### Morphology of Vagal Afferent Visceral Endings

A more specific vagal afferent classification has been made based on morphological specialisation of vagal afferent endings in the gut wall. This approach distinguishes vagal afferent populations into three subtypes, namely the intraganglionic laminar endings (IGLEs), intramuscular arrays (IMAs) and mucosal afferents (Berthoud and Powley, 1992).

IGLEs have been found in the myenteric plexus, forming fine laminar or aggregate puncta surrounding the myenteric ganglia (Fox et al., 2001a). These IGLEs have been shown to be distributed, without any obvious regional specialisation, across the GI tract (Berthoud et al., 1997). In contrast, IMAs are located in discrete locations, such as the longitudinal (longitudinal IMA) and circular (circular IMA) muscle sheets in the sphincter regions and the stomach (Powley et al., 2012, 2013, 2014, 2016). Circular IMAs are predominant in the lesser curvature while longitudinal IMAs are abundant in the greater curvature region of the stomach (Powley et al., 2016). Despite the telodendria-like classical structure, IMA nerve endings also display specialisations, such as modification of arbours, density, and depth of nerve ending penetration, in particular regions of the GI tract. For instance, IMAs that innervate the pyloric sphincter form an annulus ring (Powley et al., 2014), whilst a shorter and denser IMA population has been observed in the sling and clasp of the lower esophageal sphincter (Powley et al., 2013, 2016).

The mucosal layer of the gut wall is also innervated by vagal projections known as mucosal afferents. These endings penetrate into the lamina propria where they may have contact with epithelial cells but not to the luminal content (Wank and Neuhuber, 2001; Powley et al., 2011). Similar to IMAs, mucosal afferents display specialised substructures with regards to its innervated organs. For instance, four classes of mucosal afferent endings have been identified in the upper cervical oesophagus of the rats (Wank and Neuhuber, 2001). In the small intestine, studies in rats have revealed two distinct substructures innervating the crypts and villi of the proximal small intestine, respectively (Berthoud et al., 1995; Powley et al., 2011; Serlin and Fox, 2020). Whilst these previous studies examined distinct regions of the small intestine, accounting for only about 1-2% of the whole length, a recent study by Serlin and Fox described these endings quantitatively and qualitatively for the entire length of the mouse small intestine (Serlin and Fox, 2020). Furthermore, a mucosal afferent ending specialisation was also observed in the antral gland of the stomach (Powley et al., 2011).

Ending specialisation and the existence of distinct substructures in different regions of the GI tract suggest a specific vagal afferent function. However, it is difficult to prove this premise *in vivo* since separation of each ending is required. To date, muscular and mucosal afferents can be distinguished using a differential retrograde tracing methodology (Young et al., 2008), however, no technical approach has been developed to selectively trace IGLEs or IMAs. A study has established G-protein coupled receptor (GPCR) profiles of GI vagal afferents in the muscular and mucosal layer of the gut wall (Egerod et al., 2018). Although IGLEs and IMAs were not clearly distinguished in this study, two subtypes of vagal afferents, with distinct GPCR profiles, were described in the muscular layer (Egerod et al., 2018). In addition, Bai et al. (2019) has recently profiled and characterised GI vagal afferents based on the correlation between the morphology of the afferent endings and genetic marker expression that revealed distinct populations of IGLE in the stomach (*Glp1R*^+^), IGLE in the small intestine (*Oxtr*^+^), mucosal afferents in the pyloric antrum (*Sst*^+^/*Gpr65*^+^), mucosal afferents in the lesser curvature of the corpus (*Calca*^+^), IMAs near gastric antrum and large intestine (*Calca*^+^), mucosal endings in the small intestine (*Gpr65*^+^), and mucosal afferents in the intestinal villi (*Vip*^+^/*Uts2b*^+^). Therefore, there is potential for this knowledge to be adapted to develop a molecular-based targeting approach to differentiate the physiological roles of distinct vagal afferents population in the GI tract.

Although the focus of this review is vagal afferent innervation, spinal afferents also project to the upper GI tract. Eight distinct ending subtypes have been identified in the stomach after injection of dextran biotin in DRG T8 – T12 (Spencer et al., 2016a). However, their individual functions are still unclear. Spinal afferents are predominantly known for their function in sensing noxious stimuli. Nevertheless, these fibres also detect innocuous mechanical and chemical stimuli that may account to gut physiology (Schwartz and Gebhart, 2014; Spencer et al., 2014). Furthermore, it is possible that spinal afferents contribute to appetite regulation, with a study suggesting the involvement of spinal afferents in hypoglycemic detection in the portal vein (Fujita and Donovan, 2005). Further studies are required to establish whether gastric spinal afferents play a role in food intake regulation.

### Physiological Functions of Vagal Afferent

Vagal afferent endings in the GI tract serve as receptive fields towards various type of stimuli, such as mechanical, chemical and thermal. Based on the response, GI vagal afferents are classified into three major classes, namely mechanoreceptors, chemoreceptors and thermoreceptors.

#### Vagal Afferent Mechanoreceptors

Vagal mechanosensing is an important component in the physiology of digestive function that is prominent in the stomach. This perception is important for the maintenance of energy homeostasis as well as gut motility and secretion, by detecting physical changes during ingestion and digestion of food. Vagal mechanosensors are located in the mucosal and muscular layer of the gut wall, sensing tension and tactile stimuli (Grundy and Scratcherd, 2011). Based on their response to different types of mechanical stimuli, GI vagal afferents are categorised into tension, mucosal, tension-mucosal receptors, and stretch receptors (Phillips and Powley, 2000; Brookes et al., 2013).

##### Tension receptors

Tension-sensitive vagal afferents were first described by Iggo in 1955, termed as “in-series” tension receptors following their response to passive distension and active contraction of the smooth muscle (Iggo, 1955). Since then, extensive electrophysiological studies have been conducted to characterise these receptors in different GI organs of various species. In general, tension receptors are identified as slowly adapting, low threshold mechanoreceptors which respond to circular tension and high intensity mucosal stroking (Page and Blackshaw, 1998; Page et al., 2002). In 2000, the first evidence correlating tension receptors to a specialised ending structure, i.e., IGLE, was established (Zagorodnyuk and Brookes, 2000). Genetic-based studies have shown that a population of IGLEs in the stomach expresses glucagon-like peptide 1 receptor (GLP1R) (Williams et al., 2016; Bai et al., 2019). This subtype is specifically activated by mechanical distension *in vivo* (Williams et al., 2016), reinforcing the possible function of IGLEs as a mechanotransduction site. Furthermore, a population of IGLE expressing oxytocin gene (*Oxtr*) has also been identified in the small intestine (Bai et al., 2019).

The role of tension receptors in sensing distension has been associated with food intake regulation. Studies in humans have shown that mechanical stretch of the stomach limits food intake by inducing satiation and satiety (Marciani et al., 2001, 2015; Feinle-Bisset, 2016). In animal models, a recent study using opto-and chemogenetic approaches in mice has demonstrated that activation of vagal GLP1R subtypes inhibits neurons expressing Agouti-related protein (AgRP neurons) in the hypothalamus and limits food intake (Bai et al., 2019). This inhibition is rapid but transient (Bai et al., 2019). Gastric tension receptors express a variety of GI hormone receptors and ion channels (Christie et al., 2018; Bai et al., 2019), suggesting interactions between neural and humoral pathways in modulating mechanosensation. For instance, Kentish et al. (2015) proposed the role of transient receptor vanilloid 1 (TRPV1) in gastric vagal afferent signalling, given the evidence of dampened tension receptor mechanosensitivity in TRPV1 knockout mouse. In addition, previous studies have shown that in high fat diet-induced obese mice, diurnal rhythms in gastric tension receptor mechanosensitivity are lost and accompanied by a loss of diurnal rhythms in food intake (Kentish et al., 2013). Further, a reduction in food intake was observed in a chronic stress mouse model where gastric tension receptor mechanosensitivity was increased (Li et al., 2019). To date, the mechanism underpinning modulation of gastric tension receptors in a broader physiological context and disease pathophysiology is unclear and remains to be determined. In addition, it has been shown that activation of mechanosensitive vagal *Oxtr* also limits food intake by inhibiting AgRP neurons (Bai et al., 2019). Interestingly, activation of *Oxtr* neurons by intestinal distension produced a rapid and sustained inhibition of AgRP neurons and significantly reduced food intake (Bai et al., 2019), suggesting a potential role of intestinal mechanosensation in the central control of food intake besides its canonical function in the intestinal brake mechanism (Alleleyn et al., 2016). Further studies are required to investigate the orchestration of feeding behaviour involving this subtype alongside gastric tension receptors and humoral pathways.

Besides vagal tension receptors, there are populations of mechanosensitive enteric neurons (ENs) that can respond to various types of mechanical stimuli and act as largely tension or tone-sensitive afferents (Furness et al., 2014; Page and Li, 2018), or length-sensitive afferents that are independent of tension or tone (Spencer and Smith, 2004; Spencer and Hu, 2020). In contrast to IGLEs, mechanosensitive ENs are activated by soma deformation and display no specific mechanotransduction sites, suggesting functional specialisation of these neurons in regulating gut motility (Kugler et al., 2015). IGLEs and ENs are located in close proximity within the myenteric plexus, however, no studies have reported the contribution of their interaction in GI mechanosensitivity (Umans and Liberles, 2018). In a broader context, several studies have proposed a role for vagal nerve and EN interactions in disease pathophysiology, e.g., Parkinson’s disease (Ulusoy et al., 2017). In humans, truncal but not selective vagotomy has been suggested to have a protective effect towards Parkinson’s disease (Liu et al., 2017; Breen et al., 2019). Furthermore, accumulation of α-synuclein, a hallmark of Parkinson’s disease, has been detected in ENs (Anselmi et al., 2018) and vagal nerves have been proposed as the key mediator for α-synuclein transport between the ENs and the brain in mice (Santos et al., 2019). α-synuclein was detected in the DMV and substantia nigra (Kim et al., 2019; Van Den Berge et al., 2019) after injection of pathologic α-synuclein into the muscular layer of the pylorus and duodenum, suggesting gut to brain transport of α-synuclein. Evidence suggests the involvement of vagal efferents in the spread of α-synuclein (Phillips et al., 2008). However, a possible role of vagal afferents in this mechanism requires further examination, particularly given that a circuit involving vagal afferent has been identified to connect the gut and the dopaminergic neurons in the substantia nigra (Han et al., 2018).

A population of high threshold vagal mechanoreceptors have also been identified in the GI tract. Whilst low threshold vagal mechanoreceptors have been related to innocuous physiological responses (Paintal, 1953; Iggo, 1955; Blackshaw et al., 1987; Page et al., 2002) the specialised population of high threshold vagal mechanoreceptors, in the oesophagus, has been associated with nociceptive properties similar to spinal afferents (Yu et al., 2005; Kollarik et al., 2010b). However, further studies are required to reveal the mechanisms since distinct receptors and pathways may be involved.

##### Stretch receptors

To date, the idea that tension and stretch stimuli in the GI tract are detected by independent vagal mechanoreceptors is still in debate. Mechanosensitive vagal afferents in the stomach were initially described as stretch receptors (Paintal, 1954). Tension receptor vagal afferent mechanoreceptors were generally described as a homogenous population of tension-sensitive afferents that detect both muscle stretch and tension (Iggo, 1955; Phillips and Powley, 2000). Stretch and tension are two different types of forces. Stretch reflects the force needed for muscle extension or contraction, while tension is the force given to maintain muscle length (Phillips and Powley, 2000). The discovery of two distinct endings in the muscle layer of the gut wall raises the possibility of the existence of independent stretch receptors (Berthoud and Powley, 1992), with Phillips and Powley proposing IMAs as stretch receptors (Phillips and Powley, 2000).

Studies have shown that IMAs interact with interstitial cells of Cajal (ICC) via synaptic connexions in the muscle layer (ICC-IM) (Powley et al., 2008). In c-Kit and steel mutant mice, lacking ICC-IMs, there was a selective loss of IMAs, whereas stomach and intestinal IGLEs remained unaltered (Fox et al., 2001b, 2002). Studies have used these mouse models to investigate IMAs function in feeding behaviour, where changes in meal patterns, marked by smaller meal size and increased meal frequency, were observed in both mouse models (Fox et al., 2002; Chi and Powley, 2003). However, there were no changes in total daily food intake. Moreover, c-Kit mice displayed increased sensitivity to CCK (Chi and Powley, 2003). This evidence suggests a potential role of IMAs in short-term feeding regulation, presumably through regulation of the gastric accommodation reflex and gastric emptying. Indeed, ICC-IMs are known to play a key role in the initiation and coordination of GI motor activity (Dickens et al., 2001). Therefore, it is plausible that the absence of this structure may lead to the disruption of gut motility which subsequently impacts feeding behaviour. However, further studies are required to establish direct evidence on this relationship in feeding behaviour. In addition, a subset of circular muscle IMAs (collateral IMAs) projecting within the myenteric ganglia and making contact with ENs in the stomach has also been identified (Powley et al., 2016). Altogether evidence suggests conjoint functions of IMAs in local and central regulation of gut motility, IMAs might facilitate communication between ICC-EN and ICC-central nervous system (CNS), or IMAs may act as primary stretch receptors sending cues to the ICCs, ENs and the CNS to regulate gut motility. However, this is highly speculative and further investigation is required.

##### Mucosal receptors

In contrast to vagal afferent endings in the muscular layer, the physiological roles of mechanosensitive mucosal afferents in the GI tract are relatively overlooked. Mucosal afferents are fast adapting, low threshold mechanoreceptors which are activated by mucosal stroking (Page and Blackshaw, 1998; Page et al., 2002). Only a few studies have focused on mucosal mechanosensation in the last three decades, where functional roles of mucosal mechanoreceptors were mainly determined based on *in vitro* study through single fibre electrophysiology (Kentish et al., 2015) or measured *in vivo* in anaesthetized animal models (Becker and Kelly, 1983). In 1983, Becker and Kelly performed gastric emptying measurements in conscious dogs with severed antral mucosal afferents (Becker and Kelly, 1983). The caveat in this approach is that the removal of the mucosal layer of the antrum involved myotomy which disrupt the muscle layer. This could be a confounding factor as other subtypes of vagal afferent mechanoreceptors also innervate the muscle layer of the gastric antrum (Powley, 2000; Bai et al., 2019). Although these studies have suggested a role of mucosal receptors in gastric emptying, by detecting food particle size, and in the regulation of the vomiting vagal reflex (Becker and Kelly, 1983; Andrews and Wood, 1988; Phillips and Powley, 2000; Kentish et al., 2015), none have directly shown the physiological role of mucosal afferents *in vivo*. Furthermore, mucosal afferents possess a diversity of morphological substructures and the ability to detect different types of tactile mechanical stimuli (Rodrigo et al., 1970, 1975a; Pedrosa et al., 1976; Wank and Neuhuber, 2001), similar to the cutaneous touch receptor characteristics (Abraira and Ginty, 2013). While this suggests that true mucosal mechanoreceptors may act as touch receptors for the viscera, the presence of polymodal (i.e., detect chemical and thermal stimuli) vagal afferent populations may contradict this premise (Iggo, 1955; Clarke and Davison, 1978; Jänig, 1996; Lennerz et al., 2007). Thus, further studies are required to clarify this deliberation.

##### Tension-mucosal receptors

In addition to vagal tension and mucosal receptors, a novel receptive field termed tension-mucosal receptor has been observed in the oesophagus of ferret (Page and Blackshaw, 1998). A study using a similar approach was conducted in mice, however, tension-mucosal receptors were not identified (Page et al., 2002). This could be due to the thinness of esophageal tissue in mice, where low intensity mucosal stroking (10 mg von Frey hair) also evokes distension and makes differentiation of tension and tension-mucosal receptors impossible (Page et al., 2002). No anatomical studies have reported the structural existence of this vagal subtype in the gut wall, although an analogue, i.e., mucosal-muscular receptor, has been described in the pelvic and sacral spinal pathway of mouse (Brierley et al., 2018). Studies have proposed that mucosal-muscular afferent endings terminate in the mucosal and muscular layer of the gut wall (Page and Blackshaw, 1998; Brierley et al., 2004), however, it has also been suggested that responses to both tension and mucosal stimuli are transduced at the subepithelial plexus (Brookes et al., 2013). Furthermore, recent studies have characterised spinal afferents in the GI tract and discovered that a single DRG neuron can provide complex endings in the mucosa, myenteric ganglia and circular muscle (Spencer et al., 2016a, 2020), suggesting that signal transduction transmitted by a single DRG neuron could be initiated in different layers of the gut wall. To date, the location of the tension-mucosal vagal afferent endings, where the transduction signal is initiated, and their roles in GI function are inconclusive and require further investigation.

#### Vagal Afferent Chemoreceptors

The role of vagal afferents in gut chemosensation is crucial. Vagal chemoreceptive fields are distributed in the mucosal lamina propria of the gut wall. This sensory nerve detects a wide range of chemical stimuli, such as gut hormones, nutrients, osmolarity and pH change (Powley and Phillips, 2004). Modulation of vagal chemoreceptor activity can occur through nutrient absorption or increasing of mucosal permeability as in leaky gut. Since mucosal afferents do not make direct contact with luminal content, the chemosensing mechanisms are facilitated by the epithelial cells of the gut wall. Early studies have identified subclasses of vagal afferent chemoreceptors based on their activation by specific nutrients, i.e., vagal glucoreceptors (Mei, 1978), amino acid receptors (Jeanningros, 1982) and fatty acids receptors (Lal et al., 2001). Furthermore, it has been shown that vagal activation is potentially mediated by gut hormones released in the presence of specific nutrients (Dockray, 2003, 2013; Raybould, 2010).

Recently, a novel chemosensitive vagal afferent subtype, expressing G protein receptor 65 (GPR65), that detects intestinal nutrients has been discovered (Williams et al., 2016). GPR65, or T cell death-associated gene 8 (TGAD8), is a proton-sensing, psychosine-sensitive, GPCR that detects extracellular pH change (Wang et al., 2004; Ishii et al., 2005). GPR65 is mainly associated with immune cells and inflammatory responses (McGuire et al., 2009). In neurons, GPR65 are expressed in the CNS and dorsal root ganglia neurons with physiological function associated with pH homeostasis (McGuire et al., 2009) and pain (Huang et al., 2007), respectively.

Vagal GPR65 has been shown to innervate the proximal intestine villi, close to the gastroduodenal junction. Activation of vagal GPR65 was exclusively evoked by food entry to the duodenal bulb and resulted in inhibition of gastric motility, limiting food entry to the small intestine (Williams et al., 2016), but has no effect on feeding behaviour (Bai et al., 2019). The entrance of food into the duodenal bulb involves tactile movement of chyme as well as osmolarity and pH change. Serotonin has been proposed as a key mediator of vagal GPR65 activation, given that these neurons are responsive to serotonin but not CCK or glucagon like peptide 1 (GLP1) (Williams et al., 2016). Serotonin has been shown to be released from the enterochromaffin cells (ECC). Mechanical pressure to the intestinal wall has been suggested as a primary trigger of serotonin release (Hansen and Witte, 2008) although chemical stimuli such as pH changes can also trigger serotonin release (Smith et al., 2006). It has been confirmed in a recent study, that mechanical stimuli can trigger the release of serotonin via a Piezo2-dependent mechanism by a population of ECC cells in the small intestine and colon (Alcaino et al., 2018). In addition to vagal GPR65, a new subpopulation of mucosal afferents, expressing *Vip/Ust2b*, was discovered to be exclusive in the intestinal villi, where activation of this population has no effect on feeding (Bai et al., 2019).

In the distal part of the intestine, vagal afferents have been identified to make a synaptic connexion with enteroendocrine cells (EECs) via a neuropod. Neuropods are axon-like, long cytoplasmic processes that project from the basolateral side of EECs establishing direct contact with vagal afferents, enteric glia, and efferent fibres in the mucosal lamina propria (Bohórquez and Liddle, 2011; Bohórquez et al., 2014, 2015; Kaelberer and Bohorquez, 2018). With the exception of somatostatin secreting EECs, neuropods appear to be a general characteristic of EECs, with basal process length varying depending on the region of the GI tract (Larsson et al., 1979; Gustafsson et al., 2006). Neuropods were first identified in peptide YY-expressing EECs (PYY-EECs), with a high prevalence in the mouse ileum and colon (Bohórquez and Liddle, 2011). A recent study has demonstrated that infusion of sugar (i.e., sucrose) evokes glutamate mediated vagal afferent firing, with EEC glutamate release through the neuropod (Kaelberer et al., 2018). This study provides the first evidence that vagal afferents and EECs establish a direct contact in nutrient sensing transduction. However, the extent of which this occurs and their functional significance for feeding behaviour remain unclear.

It is generally considered that GI hormones mediate the communication between gut epithelial cells and vagal afferents in nutrient sensing. For instance, the presence of glucose in the small intestine induces the release of serotonin and GLP1, which activates vagal afferents in the intestinal mucosa, resulting in the regulation of gastric emptying, pancreatic exocrine and intestinal fluid secretion (Raybould, 2010). Further, CCK release, triggered by the presence of fatty acids and amino acids, has been shown to activate vagal afferents and induce satiety (Dockray, 2003). The finding of a novel pathway of communication via glutamate, occurring in the presence of nutrients, raises a further question of how vagal afferents are involved in satiety regulation via this route. However, functional roles of this pathway in physiology requires further examination. One model posits that it may occur by regulating EEC sensitivity to nutrients. Neuropods have been suggested to make contact with efferent fibres since post-synaptic markers have been identified in these cells (Kaelberer et al., 2018). Although further studies are required to reveal the downstream pathway, this suggests that the nutrient sensing mechanism may occur in a complex manner. The finding of EEC-vagal afferent circuits clarifies one possible transduction mechanism of nutrient sensing in the gut. However, whether GI hormones are involved in this mechanism or act independently remains to be determined.

#### Vagal Afferent Thermoreceptors

GI vagal afferents have been suggested to play an important role as a visceral thermosensor (Jänig, 2018). A vagal thermoreceptor is described as being an unmyelinated, mechano- and chemo-insensitive neuron, located in the mucosal layer and able to sense either cold (10-36^o^C) or warm (39-50^o^C) temperature, or both in some cases (10-35^o^C and 40-50^o^C) (El Ouazzani and Mei, 1982; El Ouazzani, 1984). Some populations of vagal mechano- and chemo-receptors also show thermosensitive activity in the noxious heat or cold temperature range (Lennerz et al., 2007). Vagal thermoreceptors are thought to have a role in detecting temperature changes during ingestion that may contribute to the maintenance of body thermal regulation and/or GI protection towards noxious temperature (Jänig, 2018). However, identifying the roles of these subtypes *in vivo* is hampered by the difficulty in selectively targeting thermosensitive afferents for electrophysiological recording.

### Brainstem Projection and Neural Circuitry of GI Vagal Afferents

GI vagal afferents and their visceral endings have been extensively studied in terms of anatomy and their ability to perceive different types of stimuli (Brookes et al., 2013; Browning et al., 2017; Waise et al., 2018). However, their central circuitry is relatively unexplored. Understanding vagal afferent trafficking in the CNS is important since the same type of receptive field, in the GI tract, may have different central endings and generate different feedback responses (Waise et al., 2018). In food intake regulation, left and right vagal ganglia have been shown to terminate in distinct regions of the NTS and regulate different aspects of physiology to control food intake (Han et al., 2018). Recent studies have examined memory control and right NG-mediated reward circuit in food intake (Han et al., 2018; Suarez et al., 2018). However, the role of the left NG neurons and their central circuits, as well as interaction between neural and humoral pathways in regulating food intake require detailed investigation. Therefore, further studies are required to map neural circuits of particular vagal afferent subtypes based on their location in the gut.

## Accessing Vagal Afferent Subtypes

**FIGURE 3:**
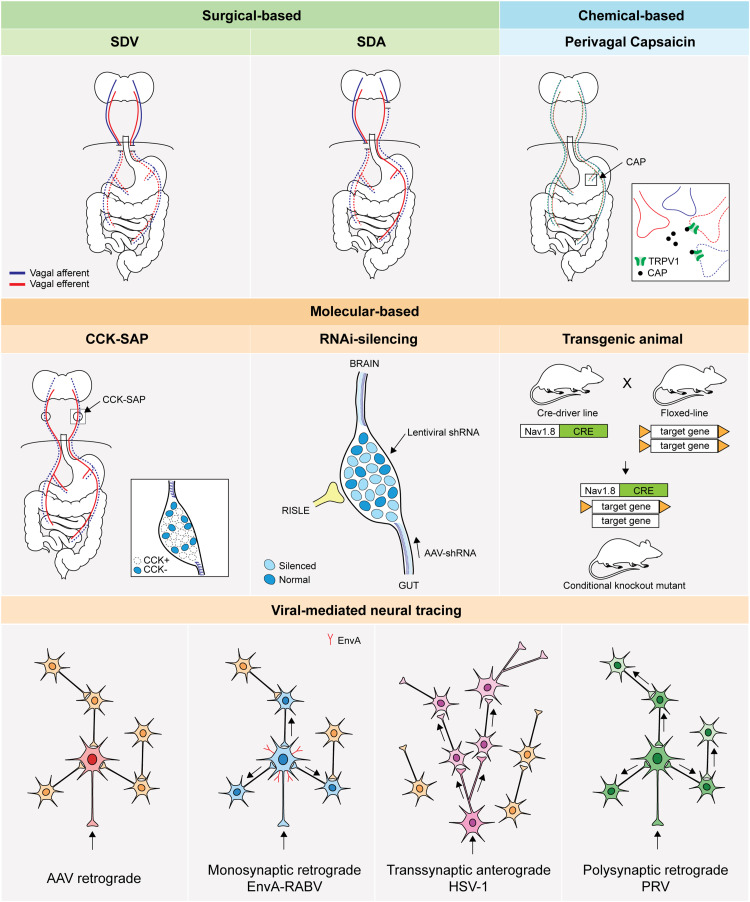
Basic principle of technical approaches to access GI vagal afferents population. An illustration of technical approaches to study vagal afferents. SDV: subdiaphragmatic vagotomy, SDA: subdiaphragmatic deafferentation, CAP: capsaicin, TRPV1: transient receptor potential vanilloid 1,CCK-SAP: cholecystokinin-saporin, RNAi: RNA interference, RISLE: RNAi-induced gene silencing by local electroporation, CRE: Cre recombinase, AAV: adeno-associated virus, EnvA: envelope protein of subgroup A avian sarcoma and leukosis virus, RABV: rabies virus, HSV-1: herpes simplex virus 1, PRV: pseudorabies virus.

Targeting vagal afferent subtypes has been a longstanding challenge considering the complexity of vagal afferent anatomical organisation, particularly their innervation in the gut (Berthoud and Neuhuber, 2000; Bai et al., 2019). The techniques currently available can be classified into three major classes: surgical, chemical and molecular-based approaches (Figure 3 and Table 2). In this review, we will discuss the basic principles, compare specificity and time course of study, and outline the advantages and limitations for each technique.

**TABLE 2:** Technical approaches to selectively target vagal afferent subtypes.

Techniques		Specificity	Advantages	Limitations	References
**Surgical**					
	SDV	Subdiaphragmatic vagal trunks	– Direct effect of vagal dysfunction	– Eliminate both vagal afferent and efferent; Surgical variability; Irreversible	Snowdon, 1970
	SDA	Subdiaphragmatic vagal afferents	– Direct effect of vagal dysfunction	– Unilateral efferent ablation; Irreversible	Norgren and Smith, 1994
**Chemical**					
	Perivagal capsaicin	TRPV1-expressing cells	– Long regeneration period (3- 5 months)	– Damage vagal efferents; Irreversible	Jancsó et al., 1977
**Molecular**					
	CCK-SAP	CCK-expressing cells	– Selectively target upper gut vagal afferents; Requires cell uptake for neurotoxic effect	– Injection must be made in the nodose ganglia; Irreversible	Diepenbroek et al., 2017
	Transgenic animals	Cell type specific	– Stable gene expression; Allows global or tissue specific mutation	– Creation and maintenance are difficult; Requires genotypic and phenotypic validation; No specific marker for NG neurons	Stirling et al., 2005; Rossi et al., 2011; Vong et al., 2011; de Lartigue et al., 2014
	siRNA	Delivery vector dependent	– Allows multiplex mRNA targeting; Allows transient or long term gene silencing effect	– Dose-dependent; Efficiency dependent on delivery method; Possible off-target gene silencing	De Lartigue et al., 2010; Kollarik et al., 2010a; Krieger et al., 2016
	**Viral tracers**				
	Adeno-associated virus	Cell specific, serotype dependent	– Non-neurotoxic, low immunogenicity; Stable and long lasting gene expression; Availability of serotypes with various directional transport capability and tropism • e.g., AAV1, AAV9, AAVrg (retrograde), AAV.PHPs	– Small genome size limit cassette size; Lead time > 4 weeks before phenotype observation	Tervo et al., 2016; Chan et al., 2017; Han et al., 2018
	Rabies virus	Neurotropic	– Fast propagation and high level of gene expression; Low cytopathic; Large genome size; Established protocol to insert gene of interests; Monosynaptic retrograde directional transport • e.g., SAD-ΔG-EGFP, SAD-ΔG-EGFP (EnvA)	– Neurotoxic; Short time course of experiments, up to 16 days	Wickersham et al., 2007a, b; Han et al., 2018; Kaelberer et al., 2018
	Herpes simplex virus 1	Neurotropic	– Fast propagation and high level of gene expression; Anterograde transsynaptic directional transport. • e.g., H129-ΔTK-TT	– Neurotoxic; Short time course of experiments, up to 5 days; Delayed retrograde transport (HSV129)	Lo and Anderson, 2011; Wojaczynski et al., 2015; Han et al., 2018
	Pseudorabies virus	Neurotropic	– Fast propagation and high level of gene expression; Retrograde transsynaptic directional transport. • e.g., PRV512, PRV 614	– Neurotoxic; Short time course of experiments, up to 5 days	Smith et al., 2000; Banfield et al., 2003; Han et al., 2018

### Surgical-Based Approaches

Vagotomy is a surgical based approach that removes vagal innervation by cutting the nerve fibres. Dragstedt developed vagotomy as a treatment for peptic ulcer patients in 1943 (Dragstedt and Owens, 1943). The cutting position was initially made on the anterior and posterior vagal trunks using a transthoracic approach (supradiaphragmatic truncal vagotomy) (Dragstedt and Owens, 1943). Since then it has been continuously refined into transabdominal subdiaphragmatic truncal vagotomy, selective vagotomy and highly selective vagotomy (Crile, 1947; Johnston and Goligher, 1976). Peptic ulcer symptoms were improved following supradiaphragmatic truncal vagotomy, however, abnormalities in gastric motility, emptying and secretion were observed (Woodward, 1987). These phenomena have drawn interest on the importance of vagal nerves in GI function and regulation of food intake.

In principle, vagotomy diminishes bidirectional signal traffic between the gut and the brain since both vagal afferent and efferent fibres are excised. Consequently, it becomes difficult to differentiate between the sensory and motor function. In 1970, Snowdon (1970) employed subdiaphragmatic vagotomy (SDV) in a rat model and proposed a role of vagal afferents in peripheral control of food intake. Shortly after, determination of vagal sensory and motor responses was conducted by measuring gastric content and gastric emptying rate respectively, in rats that underwent SDV (Snowdon, 1970). In 1974, Powley and Opsahl demonstrated that SDV neutralised the effect of ventromedial hypothalamus (VMH) lesion to induce obesity in rats but not in genetically obese Zucker rats (Opsahl and Powley, 1974), suggesting a role for the vagus nerve in body weight maintenance. Further examination of vagal afferent and efferent functions was performed by comparing SDV to atropine sulphate treatment. Atropine is an anticholinergic/antimuscarinic agent that abolishes parasympathetic tone via competitive binding to cholinergic or muscarinic receptors (Broadley and Kelly, 2001). This substance is known to inhibit vagal efferent activity (Mittal et al., 1997; Yamakawa et al., 2015). However, studies have shown incomplete motor function blockade due to a non-cholinergic efferent pathway (Powley et al., 1978) and unspecific efferent inhibition in other ganglia (Feldman et al., 1979). Taken together, determination of sensory and motor function using this approach remains problematic.

An improved division of vagal afferent and efferent function using a surgical approach was made in 1994 when subdiaphragmatic vagal deafferentation (SDA) was established in a rat model (Norgren and Smith, 1994). It is known to be the most complete surgical-based vagal deafferentation to date. SDA removes all subdiaphragmatic vagal afferents but leaves 50% of efferent fibres intact by severing at the intracranial vagal afferent or efferent rootlets through a ventral approach (Norgren and Smith, 1994). A similar technique using a dorsal approach, developed earlier, was used to study the role of vagal afferents in the small intestine (Walls et al., 1995).

Completeness of vagotomy can be confirmed using various methods dependent on the type of surgery. Subsets of physiological and anatomical parameters, such as response to insulin, the presence of gastric stasis and impairment of the vago-vagal reflex, were often used to validate the loss of vagal function pre-mortem (Louis-Sylvestre, 1983). After the discovery that the CCK satiety effect is facilitated by gastric vagal branches (Smith et al., 1981), later studies predominantly used a CCK-induced satiety test, administered intraperitoneally at low doses (1 – 6 μg/kg) to validate the success of vagotomy (Moran et al., 1997; Ferrari et al., 2005; Powley et al., 2005; Suarez et al., 2018). In addition, a retrograde tracing protocol using true blue has been developed to validate total and selective vagotomy post-mortem (Powley et al., 1987). This technique provides a complete anatomical evaluation of vagotomized vagal branches and permits validation to the majority of abdominal vagal branches. Furthermore, it has been reported that regeneration may occur following vagotomy. In mice, vagal fibres reinnervate the stomach starting at week 4 post-vagotomy and achieve normal density at week 16 in the corpus, with the optimum time-frame for physiological observation within 8 weeks after vagotomy (Powley et al., 2005). On the other hand, incomplete restoration of vagal afferent innervation in the smooth muscle of rats was observed at 18 weeks (Phillips et al., 2000).

Both SDV and SDA are well-established techniques in rat, however, there are less studies using these techniques in mouse models. Mice undergoing bilateral SDV have been shown to survive for at least two weeks post-surgery (Iwasaki et al., 2015; Yoshii et al., 2017). However, a study has reported the lethality of this procedure in mice due to gastric distension and pyloric stenosis (Dezfuli et al., 2018). Heineke-Mikulicz pyloroplasty has been used to ameliorate pyloric stenosis and increase mouse survival (Dezfuli et al., 2018), however, this may affect the physiological response of the animal. These contradictive outcomes suggest individual variation of the surgical approach.

### Chemical-Based Approaches

Capsaicin (CAP) is a pungent component of *Capsicum* which was isolated in 1876 (Thresh, 1876). CAP has been widely used as an analgesic due to its anti-nociceptive properties, known as the capsaicin desensitisation phenomena (Szolcsányi, 2014). In 1977, Jancsó discovered detrimental effects of CAP on primary sensory neurons of neonatal and adult rats (Jancsó et al., 1977). This study revealed the selective action of CAP in a specific population of primary sensory neurons in the DRG and trigeminal ganglia, referred to as CAP-sensitive sensory neurons. This finding also demonstrated two mechanisms of action of CAP, a short-term excitatory and a long-term neurotoxic effect (Holzer, 1991). However, the molecular mechanism of how CAP generates these effects was unclear at that time.

The excitatory mechanism of CAP was revealed when a capsaicin receptor, termed vanilloid receptor subtype 1 (VR1), was discovered in 1997 (Caterina et al., 1997). This receptor is now known as the transient receptor potential VR1 (TRPV1) channel (Montell et al., 2002), a calcium permeable, non-selective cation channel, that can be activated by numerous agents, e.g., noxious heat, protons, divalent cations (i.e., Mg^2+^ and Ba^2+^), exogenous and endogenous TRPV1 agonists, animal toxins and plant secondary metabolites (Caterina et al., 1997; Szolcsányi, 2014; Yang and Zheng, 2017; Christie et al., 2018). CAP binds to the S3-S4 transmembrane regions of the TRPV1 channel and stabilises TRPV1 opening via a “pull and contact” interaction of the S4-S5 linker and vanillyl group (Yang et al., 2015), which increases membrane permeability to cation influx and induces depolarization in a concentration and exposure length dependent manner (Caterina et al., 1997; Chung et al., 2008; Szolcsányi and Sandor, 2012). Furthermore, it has been shown that long-term exposure of capsaicin to HEK293-expressing VR1 cells, DRG and NG neurons induces cell death (Caterina et al., 1997; Czaja et al., 2008). Intracellular acidosis, calcium overload and mitochondrial swelling, due to prolonged opening of TRPV1 channels, have been proposed as underlying mechanisms (Szolcsányi and Pinter, 2013; Chiang et al., 2015).

Systemic injection or application of CAP on vagal afferent visceral endings have been shown to cause neurodegeneration of GI vagal afferents and diminish their sensory signalling (Ritter et al., 1988; Czaja et al., 2008). Commonly, peripheral application of 1% CAP is used to induce vagal afferent lesion in a specific GI organ (Browning et al., 2013). Perivagal CAP has a slower regeneration speed giving a longer experimental time-frame compared to surgical based vagotomy. Studies in rodents have shown a regeneration period of 3 – 5 months post CAP treatment with pronounced loss of vagal neurons within 30 days (Czaja et al., 2008; Gallaher et al., 2011). Since vagal afferents are not the only cells affected by CAP, mineral oil is commonly used to prevent unspecific lesion (Ritter et al., 1988; Patterson et al., 2003). Despite milder intervention and the ease of application compared to surgical based methods, result interpretation should be considered with caution since CAP also destroys vagal efferents in the DMV following perivagal CAP treatment (Browning et al., 2013).

### Molecular-Based Approaches

#### CCK-SAP

In 2017, a novel molecular-based vagal deafferentation technique targeting upper GI tract using CCK conjugated saporin (CCK-SAP) was established. Previously, CCK-SAP has been demonstrated to successfully induce lesion in rostral ventromedial medulla neurons expressing CCK receptor (Zhang et al., 2009). More specific to this review, Diepenbroek et al. (2017) used CCK-SAP to induce neural lesion of VAN in the NG. It has been shown that CCK-SAP ablates ∼80% of muscular and ∼61.7% of mucosal vagal afferents in the upper GI tract of rats and, importantly, leaves the efferent fibres intact.

Cellular selectivity of CCK-SAP action is dependent on CCK, a regulatory peptide hormone in the GI tract, primarily secreted by I-cells and widely known for its function as a satiety hormone (Rehfeld, 2017). CCK consists of 33 amino acids (AA) derived from 115 AA prepro-CCK (Deschenes et al., 1984). Biologically active CCKs emerge in various molecular forms, such as CCK-58, CCK-39, CCK-33, CCK-22, sulphated and unsulphated CCK-8 and CCK-7, CCK-5 and CCK-4 (Noble et al., 1999). CCK-SAP utilises sulphated CCK-8 (CCK8S) as conjugate, which is the predominant structure of the biologically active CCK found in the brain (Schneider et al., 1979).

CCK binds to CCK receptor (CCKR), a member of the GPCR superfamily consisting of a 7 transmembrane domain, to enter the cell. As a GPCR, CCKRs undergo endocytosis following ligand binding, forming a coated vesicle that is transported to the endosome where CCKRs can be recycled back to the plasma membrane or degraded in the lysosome (Koenig and Edwardson, 1997; Weinberg and Puthenveedu, 2019). CCKRs are classified into CCKR-A (CCK_1_) and CCKR-B (CCK_2_) based on their affinity to sulphated and amidated CCK (Noble et al., 1999). CCK_1_ has a high affinity to sulphated/amidated CCK while CCK_2_ exhibits no preference towards sulphated/non-sulphated CCK. Instead, CCK_2_ binds to gastrin and is commonly referred to as the gastrin receptor. CCK-SAP demonstrates no significant difference in affinity to CCK_1_ and CCK_2_ (Diepenbroek et al., 2017), suggesting neurotoxic effects will occur in cells expressing both receptors. Since CCKRs are widely distributed across the GI tract and nervous system, location of injection becomes a key determinant of CCK-SAP selectivity.

SAP is a type I ribosome inactivating protein (RIP) which causes cell apoptosis, when internalised, due to the impairment of protein synthesis and DNA fragmentation (Stirpe et al., 1983; Bergamaschi et al., 1996; Bagga et al., 2003). Although a small amount of SAP can be internalised via pinocytosis, a conjugate is required for an effective internalisation and cytotoxic effect, since SAP lacks a lectin binding site that facilitates endocytosis (Stirpe et al., 1992). Many neurotoxins have been made by pairing SAP with various conjugates (substance P, isolectin B4 and neuropeptide Y) to selectively ablate neuronal cell populations in the brain (Wiley and Kline, 2000; Wiley, 2001). Besides glycoproteins and neuropeptides, monoclonal antibodies (OX7, 192 IgG, and anti-dopamine beta hydroxylase) have been utilised as conjugates to generate SAP immunotoxins (Wiley and Kline, 2000). These immunotoxins and lectin-toxins exhibit retrograde axonal transport capability, or suicidal transport, via fast axonal transport mediated by microtubules (Wiley and Kline, 2000). However, these properties have not been reported for neurotoxins, including CCK-SAP.

Recent publications have demonstrated the feasibility of CCK-SAP to be used with other techniques to target vagal afferents. For instance, this technique was performed in combination with viral mediated neural tracing in mice to reveal the gut-brain axis in reward mechanisms (Han et al., 2018). Further, CCK-SAP has also been utilised to differentiate vagal sensory and motor signalling in memory control, whilst demonstrating CCK-SAP superiority compared to SDA (Suarez et al., 2018). Besides neuropsychological features, CCK-SAP provides an option to examine vagal function in homeostatic control of appetite. VANs express both CCKRs, with CCK_1_ more abundant than CCK_2_ (Moriarty et al., 1997). Studies have shown CCK_1_ localization with receptors of orexigenic hormones (ghrelin (Date et al., 2005; Burdyga et al., 2006b), orexin-A (Burdyga et al., 2003) and melanin concentrating hormone (Burdyga et al., 2006a), anorexigenic hormones (PYY (Burdyga et al., 2008), GLP-1 (Williams et al., 2016), and leptin (Burdyga et al., 2002; Li et al., 2011), as well as TRPV1 channels (Burdyga et al., 2006b) and cannabinoid CB1 receptors (Burdyga et al., 2004). Thus, lesioning CCK_1_-positive nodose neurons also impairs satiety signals trafficking between the gut and the brain mediated by these receptors. Furthermore, it is likely that regeneration of vagal afferent fibres may occur after CCK-SAP ablation. In the original study, the blunted effect of CCK-induced satiety was still present 12 weeks after CCK-SAP treatment (Diepenbroek et al., 2017), suggesting vagal function has not recovered within this period. Given that CCK-SAP destroys neuronal cell bodies similar to CAP, it is possible that the regeneration process happens at a slow rate. However, further histological studies are required to examine the regeneration time course.

#### Cre/LoxP System

The Cre/*loxP* system plays a vital role in technical advances of vagal afferent targeting. Discovered in 1981, Cre is a 38 kDa, site-specific tyrosine recombinase, isolated from bacteriophage P1 that facilitates double stranded DNA (dsDNA) recombination by targeting *loxP*, a 34 base-pair (bp) sequence, comprising of two identical 13 bp inverted repeats, separated by a 8 bp spacer region (Sternberg and Hamilton, 1981; Hoess et al., 1982). The mechanism of Cre-mediated recombination will not be discussed since it has been extensively reviewed elsewhere (Lee and Sadowski, 2003; Van Duyne, 2015). Since position, orientation and type of *loxP* determine the final outcome of Cre-mediated recombination (Hoess et al., 1984; Abremski and Hoess, 1985), several *loxP* mutants were generated to improve control of gene expression and feasibility to insert gene of interests, such as *loxRE* and *loxLE* (Araki et al., 1997), *lox511* (Schnutgen et al., 2003), *lox2272* and *loxFAS* (Saunders et al., 2012). Schnutgen et al. developed a flip excision (FLEx) switch system with *lox511* which was adapted by Saunders et al. to create Cre-on and Cre-off recombinant adeno-associated virus with *lox2272* and *loxFAS* (Schnutgen et al., 2003; Saunders et al., 2012; Saunders and Sabatini, 2015).

Cre has been shown to effectively facilitate DNA recombination in prokaryotic cells (Sternberg and Hamilton, 1981), yeast (Sauer, 1987), mammalian cells (Sauer and Henderson, 1988) and rodent models. To increase spatiotemporal control of Cre activity, different strategies employing an inducible system were created, e.g., tamoxifen-inducible Cre (Cre-ER^T^, Cre-ER^T1^ and Cre-ER^T2^) (Feil et al., 1996, 1997) and tetracycline-dependent Cre-expression (Tet-On/Tet-Off system) (Gossen and Bujard, 1992; Gossen et al., 1995; Schönig et al., 2002). Tamoxifen is a synthetic agonist of the oestrogen receptor which is converted into its derivatives in the liver by cytochrome P450 that can be administered via oral, subcutaneous or intraperitoneal routes at different doses and forms (Goetz et al., 2008; Jahn et al., 2018). On the other hand, a tetracycline analogue (doxycycline) can be administered via oral (Saam and Gordon, 1999; Lindeberg et al., 2002), intraperitoneal and local injections (Utomo et al., 1999). Further, it is important to note that tamoxifen and doxycycline may introduce confounding factors that should be considered in designing experiment (Moullan et al., 2015; Hammad et al., 2018).

#### Transgenic Animal Models

The use of transgenic animal models marked the entry of molecular tools to study vagal afferents. In the early 2000s, developmental studies using neutrophin-4 (NT-4) and c-Kit mutant mice demonstrated specific aberration of vagal afferent subtypes in the GI tract (Fox et al., 2001a, b). These mouse models exhibit changes in meal patterns that suggest a role of vagal afferents in short-term feeding regulation. NT-4 is a potent survival factor of CNS and peripheral nervous system (PNS) neuronal development (Huang and Reichardt, 2001). Mice lacking NT-4 exhibit a 55% reduction in size and number of neurons in nodose-petrosal and geniculate sensory ganglia (Conover et al., 1995; Liu et al., 1995). An anterograde labelling study using wheat germ agglutinin-horseradish peroxidase (WGA-HRP) has demonstrated major loss of IGLEs in the duodenum and ileum (90 and 81%, respectively) while the stomach innervation remained unaltered (Fox et al., 2001a). On the other hand, heterozygous c-Kit mutant mice exhibit deficiency of IMA formation in the forestomach (Fox et al., 2001b). This model is also ICC-IM deficient (Burns et al., 1996). c-Kit is a receptor tyrosine kinase, encoded by gene in white spotting (W) locus in chromosome 5 in mice (Bernstein et al., 1990). Spontaneous mutation can occur in W locus, affecting c-Kit expression and altering embryonic development and hematopoiesis (Geissler et al., 1988). c-Kit mutant mice were initially developed as a macrocytic anaemia model (Russell, 1979; Chabot et al., 1988). The absence of specific GI vagal afferent subtypes indicates that these models may be suitable for targeting IGLE or IMA in a particular GI organ. However, apart from the studies above, to date no other studies have reported use of these transgenic animals to investigate vagal afferent function in the gut.

Later studies predominantly utilise Cre/*loxP* technology to generate a more precise genetic modification in transgenic animals. Development of Cre-driver and Cre-dependent mouse lines are rapidly growing. However, major caveats of using this approach are the difficulties in creating, validating and maintaining the transgenic animal lines. Cre-driver and/or Cre-dependent mouse lines are exposed to the possibility of nonspecific gene expression, variability in breeding efficiency and Cre toxicity (Heffner et al., 2012). Thus, genotypic and phenotypic profiling are required to validate the transgenic animal characteristics. Such information for the majority of established transgenic mouse lines can be obtained from databases, e.g., CrePortal^1^ (Heffner et al., 2012). Studies by Fox et al. (2013) and Biddinger and Fox (2014) were the first to use the Cre/*loxP* system to selectively manipulate vagal afferents and investigate their function in feeding behaviour. They targeted nerve growth factor genes that control vagal sensory development in GI smooth muscle and demonstrated changes in meal size without vagal efferent damage. This strategy have been discussed in detail previously (Fox, 2006). Furthermore, transgenic animal models available for anatomical tracing for visualisation of the gut-brain axis has been recently reviewed (Udit and Gautron, 2013). Hence, we will focus on the frequently used Cre-driver line for parental backgrounds to study vagal afferent function, i.e., Na_v_1.8-Cre, Vglut2-Cre and Phox2b-Cre.

Na_v_1.8 is a tetrodotoxin resistant voltage-gated sodium channel particularly expressed in peripheral sensory neurons (Akopian et al., 1996). Studies have reported selectivity of Na_v_1.8 expression in the small-diameter dorsal root ganglia, trigeminal neurons and, importantly, ∼80% of NG neurons (Stirling et al., 2005; Gautron et al., 2011). Stirling et al. (2005) developed a heterozygous Na_v_1.8-Cre line mouse model which expresses Cre recombinase under Na_v_1.8 promoter regulation (Stirling et al., 2005). Genetic tracing of Na_v_1.8-Cre revealed the predominant innervation of Na_v_1.8 neurons in the mucosa and myenteric plexus of the stomach and small intestine, where IGLE morphology but not IMA was observed in the muscle layer (Gautron et al., 2011). This should be taken into consideration if individuals are aiming to understand a specific population of vagal afferent. Nonetheless, Na_v_1.8-Cre has become a versatile parental background to generate models for understanding the role of vagal afferents in food intake control. For instance, global knockout of Na_v_1.8 was generated to investigate vagal role in caloric intake regulation and pain sensing mechanism by crossing Na_v_1.8-Cre with a mouse line carrying floxed-STOP-DTA (Abrahamsen et al., 2008; Udit et al., 2017). Further, de Lartigue et al. (2014) examine the role of leptin receptor in vagal afferents by selective knockout of leptin receptor in Na_v_1.8 neurons (Na_v_1.8/LepR^fl/fl^). Additionally, other transgenic animals, such as the Na_v_1.8 null model (Akopian et al., 1999), BAC-Na_v_1.8-Cre (Agarwal et al., 2004), and heterozygous Na_v_1.8 Cre-ER^T2^ (Zhao et al., 2006) have also been developed.

Vesicular glutamate transporter (VGLUT), a membrane-bound protein facilitating glutamate trafficking into presynaptic vesicles, is known as a marker for glutaminergic neurons. There are three transporters that have been characterised so far, namely VGLUT_1_ (Bellocchio et al., 2000; Takamori et al., 2000), VGLUT_2_ (Aihara et al., 2000; Bai et al., 2001; Fremeau et al., 2001) and VGLUT_3_ (Fremeau et al., 2002; Schafer et al., 2002). The functional role of VGLUT_2_ is associated with autonomic and sensory pathways (Varoqui et al., 2002). In the CNS, expression of VGLUT_2_ mRNA is distinct to thalamus, brainstem and deep cerebellar nuclei, and transiently expressed in developing hippocampal neurons (Fremeau et al., 2001, 2004). Whereas in the PNS, expression of VGLUT_2_ has been reported in DRG (Scherrer et al., 2010) and vagal afferents (Tong et al., 2001; Corbett et al., 2005). There are several VGLUT Cre-driver lines, such as BAC-VGLUT_2_-Cre (Borgius et al., 2010) and V*glut2*-ires-Cre (Vong et al., 2011). These models have been used to differentiate vagal sensory and motor neurons functions (Williams et al., 2016; Han et al., 2018).

Paired-like homeobox 2 (*Phox2*) genes (e.g., *Phox2a* and *Phox2b*), encode homeodomain transcription factors that are essential for sympathetic, parasympathetic and ENs development (Pattyn et al., 1999; Brunet and Pattyn, 2002). Whilst *Phox2a* is responsible for neuron survival (Valarche et al., 1993), *Phox2b* is vital for cranial ganglia differentiation to acquire visceral neuron characteristics (D’Autreaux et al., 2011). *Phox2b* expression is observed in the CNS (visceromotor, branchiomotor, NTS, AP, non-adrenergic centres, and serotonergic neurons) and PNS (epibranchial and autonomic ganglia)(D’Autreaux et al., 2011). The absence of *Phox2b* resulted in the absence of other CNS and PNS neurons, while epibranchial ganglia were still present although atrophic (Morin et al., 1997; Pattyn et al., 1997; D’Autreaux et al., 2011). *Phox2b-*Cre mouse line has been widely used to target vagal afferents (Rossi et al., 2011; Scott et al., 2011; Liu et al., 2014; Kaelberer et al., 2018). Characterization of *Phox2b*-Cre illustrates limited Cre expression in the PNS (Rossi et al., 2011) where cre activity was detected in NG and second order visceral sensory neurons in the NTS but absent in other parasympathetic and sympathetic ganglia (Liu et al., 2014). Indeed, expression of *Phox2b* is known as a marker to differentiate nodose and jugular neurons (Kupari et al., 2019). While this provides high specificity to target nodose neurons *in vitro*, alteration in other *Phox2b*-expressing cells *in vivo* can be a confounding factor.

#### RNA Interference-Mediated Gene Silencing

RNA interference (RNAi) is a native regulatory mechanism that controls gene expression via post-transcriptional gene silencing in multicellular organisms (Fire et al., 1998; Elbashir et al., 2001). RNAi-mediated gene silencing causes a hypomorphic effect, a reduction but not a complete loss of phenotype. Two small regulatory, double stranded RNAs (dsRNAs), known as small interfering RNAs (siRNAs) and microRNAs (miRNAs), are important for the initiation of RNAi (Carthew and Sontheimer, 2009). A dsRNA-processing enzyme called dicer, converts these small RNAs into shorter fragments (21-23 bp). These short fragments bind to the Argonaute protein, forming siRNA/miRNA-induced silencing complex (si/miRISC) that recognises targeted gene messenger RNAs (mRNAs) and induces degradation (Mello and Conte, 2004; Setten et al., 2019). While miRNAs are mainly produced by the cells, the source of siRNAs can be endogenous (noncoding dsRNAs) or exogenous (synthetic siRNAs). Further, the mechanism of how siRNAs and miRNAs induce RNAi is distinct. Each siRNA has a complementary sequence of a specific mRNA that guides the binding of siRISC precisely and initiates mRNA cleavage (Lam et al., 2015). Conversely, miRNAs are less specific since one miRISC can identify several different mRNAs, and induce gene silencing via translational repression and mRNA destabilisation, followed by mRNA cleavage (Fabian and Sonenberg, 2012; Jonas and Izaurralde, 2015).

Synthetic siRNA has been widely used to specifically knockdown gene expression in vagal afferents. In general, exogenous siRNA can be introduced into the cells as siRNA particles, or as a sequence embedded into a viral genome and endogenously expressed by the cells as siRNA precursors. As a foreign molecule, siRNA often requires a structural modification or a carrier for efficient transport into the cells (Roberts et al., 2016). Different methods, such as lipofectamine for naked siRNA (Heldsinger et al., 2012) and magnetofection for nanoparticle-conjugated siRNA (De Lartigue et al., 2010), have been used to deliver siRNA particles in VAN cell cultures. In addition, a delivery method using local electroporation (RISLE) was developed to facilitate siRNA delivery *in vivo* in the brain (Akaneya et al., 2005), and this approach has been successfully replicated to deliver siRNA into NG (Zhou et al., 2010). A lead time of 3-6 days is required for gene silencing to occur with knockdown effects lasting for 2 weeks (Akaneya et al., 2005). It is important to note that direct introduction of exogenous siRNA particles results in a transient gene silencing effect.

Currently, viral vectors are predominantly used to deliver siRNA precursors in a form of short-hairpin RNA (shRNA) *in vitro* and *in vivo*. The use of viral vectors increases transport efficiency and provides alternatives for a stable silencing effect, given that they utilise a natural mechanism to enter the cells and to express their genome using the host system. Lentivirus is predominantly used as vector to transfer siRNA (Sakuma et al., 2012). For instance, Krieger et al. (2016) delivered GLP1R shRNA to mouse NG, resulting in 80% silencing of GLP1R expression using lentiviral vector measured 20 days after injection. *In vitro*, lentiviral vector has been shown to efficiently induce gene silencing in nodose neuron cell culture with 3-4 days incubation preceding the observation (Heldsinger et al., 2012). In addition, Kollarik et al. (2010a) used adeno-associated virus (AAV2/8) to deliver TRPV1 shRNA into NG from vagal afferent endings in the guinea pig oesophagus, efficiently silencing TRPV1 expression. Besides its specificity, siRNA-induced gene silencing provides flexibility to target different protein isoforms by introducing a combination of siRNAs (Wang et al., 2017). This allows simultaneous gene silencing that could be beneficial to understand cellular pathways. However, the silencing effect of siRNA-induced RNAi is dose-dependent. Insufficient amounts of siRNA may lead to inadequate silencing, whereas, excessive amounts of siRNA may induce non-specific gene silencing that alters phenotype (Jackson and Linsley, 2010).

#### Viral-Mediated Neural Tracing

Neural tracing is a classic method to map anatomical distribution of vagal afferents from their cell bodies or axon terminals. An important feature of neural tracing is the ability to selectively target a specific vagal afferent population based on their location in the gut or the brain, by injecting anterograde or retrograde tracer. This allows a specific examination of the functional properties of a particular vagal afferent population. A variety of biochemical-based tracers, such as cholera toxin subunit B, fluorogold, *Phaseolus vulgaris*-leucoagglutinin, WGA-HRP, lipophilic carbocyanine dyes, and variants of dextran amines, have been utilised to visualise the vagal gut-brain axis (Berthoud and Powley, 1992; Neuhuber et al., 1998; Powley, 2000; Young et al., 2008; Powley et al., 2013). In recent years, development of neurotropic viral vectors has significantly progressed, leading to ground breaking findings on vagal afferent subtypes and their functions in the gut. Here, we will focus on adeno-associated virus, rabies virus, and herpes viruses. We exclude lentivirus-based neural tracers since they mainly target motor neurons (Hirano et al., 2013; Sheikh et al., 2018).

##### Adeno-associated virus

Adeno-associated virus (AAV) is a 25 nm, non-enveloped, single stranded DNA (ssDNA) virus, isolated from Adenovirus preparation (Atchison et al., 1965; Balakrishnan and Jayandharan, 2014). Recombinant AAV (rAAV) is generated by replacing life cycle genes (i.e., *Rep, Cap*, and *aap*) (Sonntag et al., 2010; Balakrishnan and Jayandharan, 2014) between two T-shaped inverted terminal repeat (ITR) sequences with the gene of interests. The total length of rAAV genome should not exceed the wild type AAV genome (∼5 kbp) to avoid reduction in transduction efficiency (Wu et al., 2010) although strategies to deliver large transgene have been developed (McClements and MacLaren, 2017). rAAVs are highly favourable neural tracers, given their nature as a non-pathogenic, low immunogenic, and self-replication defective virus (Weitzman and Linden, 2011) makes rAAVs less neurotoxic compared to other viral vectors. This vector also provides stable gene expression without transgene integration to the host genome.

Serotypes determine AAV tropism and transport directions as neural tracers. Currently, there are 12 AAV (1-12) serotypes with more than one hundred variants (Gao et al., 2005). The majority of native AAV serotypes have tropisms towards neurons at different degrees. However, AAV9 has been shown to profoundly transduce neurons in the CNS, PNS and enteric nervous system (Howard et al., 2008; Schuster et al., 2014). Furthermore, several serotypes (AAV1 and AAV9) also display anterograde transneuronal tracing properties (Zingg et al., 2017).

Characteristics of AAV can be modified using pseudotyping or direct evolution. Pseudotyped AAV is created by combining capsid and ITR sequences from two different AAV serotypes. Kollarik et al. (2010a) utilised this approach to develop AAV2/8 which has an improved retrograde transport capability and transduction efficiency in esophageal vagal afferents compared to AAV2, AAV2/2, AAV2/7 and AAV 2/9. Denotation of pseudotyped AAV, e.g., AAV 2/8, indicates that the virus carries genome from AAV2 and capsid from AAV8. In 2017, a novel method, namely Cre recombinase based AAV targeted evolution (CREATE), was developed (Chan et al., 2017). This technique uses a Cre/*loxP* system to generate a library of capsid sequences from one AAV serotype which then undergo *in vivo* selection, termed as direct evolution, to obtain the serotype with desired characteristics. Several robust neurotrophic AAV serotypes, i.e., AAV9-PHPb (Chan et al., 2017), AAV9-PHPs (Chan et al., 2017), and AAV2-retrograde (Tervo et al., 2016), have been developed through this method. AAV9-PHPb and AAV9-PHPs developed tropism towards CNS and PNS neurons, respectively (Chan et al., 2017). Although it has been shown that AAV9-PHPs effectively transduced DRG neurons, there has been no study reporting the capability of this serotype to transduce vagal afferents. In contrast, AAV2-retrograde (AAVrg) has shown tropism to both CNS and PNS neurons (Tervo et al., 2016; Han et al., 2018). Developed by Tervo et al. (2016), AAVrg has an enhanced retrograde transport ability in neurons. A recent study has shown that AAVrg effectively transduce vagal afferents and is transported in a retrograde manner from the gut to the NG (Han et al., 2018).

Despite its robustness, one caveat of using AAV-based neural tracers is that the lead time to observe phenotype or behavioural changes takes at least 2-6 weeks after injection. This is due to a lag time for conversion of AAV ssDNA to dsDNA and genome instability post dsDNA conversion delaying gene expression (Ferrari et al., 1996; Wang et al., 2007). Self-complementary AAV (scAAV), a double stranded DNA variant of AAV, can be used to shorten the lag time (McCarty, 2008).

##### Rabies virus

Rabies virus (RABV) is an enveloped, retrograde transsynaptic neurotrophic virus from the *Rhabdoviridae* family, with a 12 kb negative-sense single stranded RNA genome. RABV envelope protein, called rabies glycoprotein (RG) (Conzelmann et al., 1990), with interaction with its receptors compulsory for infection and propagation to occur (Morimoto et al., 2000; Lafon, 2005). In 2007, Wickersham et al. (2007a, b) developed engineered RABVs, a first order retrograde neural tracer SADΔG-EGFP and a monosynaptic retrograde neural tracer SADΔG-EGFP(EnvA). Both engineered viruses lack RG, which hampers their transsynaptic spread ability. SADΔG-EGFP can infect any neurons, however, it cannot spread outside the initially infected neuron (Wickersham et al., 2007a). This virus has been used to map neural circuits in reward pathways that receive vagal input (Han et al., 2018). In contrast, SADΔG-EGFP(EnvA) express envelope protein of subgroup A avian sarcoma and leukosis virus (EnvA) which allows specific infection on cells expressing EnvA receptor, termed as TVA (Barnard et al., 2006). This strategy has been adapted to target nodose neurons *in vitro* and ECC expressing CCK *in vivo* to reveal signal transduction between vagal afferents and neuropods in nutrient sensing (Kaelberer et al., 2018).

RABV-based neural tracers are suitable for tracing back vagal projection from its ending in the viscera or region of the CNS. However, they may not be useful for studying neurons receiving vagal inputs from the NG. RABV has a low cytopathic effect, rapid infection in the CNS and high level of gene expression (Wickersham et al., 2007a, b). With a relatively large genome size and an established protocol to generate recombinant ΔG rabies (Osakada and Callaway, 2013), any gene of interest can be inserted to achieve desired aims. However, neural tracing using RABV-based tracers is only ideal for a short term (up to 16 days) experimental time course due to the pathogenic nature of RABV (Wickersham et al., 2007a). Neurotoxic effects, marked by morphological changes (e.g., blebbing) in surviving neurons occur with prolonged incubation (Wickersham et al., 2007a).

##### Herpes viruses

Two classes of herpes viruses, namely herpes simplex virus-1 (HSV-1) and pseudorabies virus (PRV), are neurotropic viruses with transneuronal spread ability and tropism to sensory and autonomic neurons (Ugolini et al., 1989; Babic et al., 1993). Both viruses are enveloped, have a linear dsDNA genome (HSV-1:150 kbp and PRV:140 kbp), and require interaction between envelope protein and host cell surface to enter the cells and propagate (Ugolini, 2010). The major difference between HSV-1 and PRV is that only HSV-1 can infect primates (Ugolini, 2010).

Herpes viruses-based neural tracers are generally transmitted in a retrograde direction. However, a unique HSV-1 strain, called HSV-1 strain 129 (H129), displays transneuronal spread in an anterograde manner (Zemanick et al., 1991; Rinaman and Schwartz, 2004). Rinaman and Schwartz utilised H129 to map vagal input from the stomach wall to the CNS (Rinaman and Schwartz, 2004) while Krieger et al. (2018) revealed central neurons receiving vagal input from left NG in controlling brown adipose tissue thermogenesis. In 2011, a Cre-dependent H129 recombinant, called H129ΔTK-TT, was generated (Lo and Anderson, 2011). Han et al. (2018) utilised H129ΔTK-TT to map vagal outputs from the right NG and discovered vagal input to dopaminergic neurons in the substantia nigra.

PRV-based neural tracers were developed from the non-virulent PRV strain Bartha. PRV-Bartha are distinct from other retrograde tracer viruses given their retrograde transport occurs exclusively from postsynaptic to presynaptic neurons (Pickard et al., 2002). Two PRV-based tracers, PRV152 and PRV614, were generated by inserting genes encoding GFP and RFP, respectively (Smith et al., 2000; Banfield et al., 2003). These variants were used to confirm the vagal (right NG)-parabrachial-nigrostriatal circuit and its necessity in food intake regulation (Han et al., 2018).

Similar to RABV, the major concern of using HSV-1 and PRV-based neural tracers is their neurotoxicity, resulting in a short experimental time course of approximately 5 days (Brittle et al., 2004; Lo and Anderson, 2011). Since the neurotoxic effect occurs rapidly, prolonged viral incubation may induce cell death in initially infected neurons, making observation of neural circuits difficult. Furthermore, there is a possibility of unspecific infection due to local spread around the injection site (Ugolini et al., 1987). The dose of viral injection is an important factor affecting this local spread and subsequently transneuronal transfer efficiency. In addition, it is important to note that HSV129 can undergo a delayed retrograde transport, approximately 3 days after initial injection, which may confound results (Wojaczynski et al., 2015).

## Assessing Vagal Afferent Subtypes Function

### *In vivo* Modulation of Vagal Afferent Activity

The action potential (AP) is a signature of basic forces in neural activity, excitation and inhibition, which is essential for neural communication. The AP relies on ionic balance that regulates membrane potential through depolarization and hyperpolarization. Following the basic principle of an AP, we can modulate neural activity using genetically encoded optogenetic or chemogenetic tools to switch on or off the signal transmission. A recent review has discussed available optogenetic and chemogenetic tools in neurogastroenterology, with a focus on the enteric nervous system (Boesmans et al., 2018). In this review, we provide an update on the current progress in GI vagal afferents.

Optogenetic refers to the use of opsins to modulate neural activity. Opsins are a family of seven-transmembrane, light-sensitive proteins activated by light exposure at a specific wavelength. Current optogenetic tools, such as channelrhodopsins, halorhodopsins and bacteriorhodopsins are derived from microbial opsins (type I opsins). Channelrhodopsin-2 (ChR2) was the first opsin employed as an optogenetic tool. ChR2 is a light-gated proton pump, which is activated by millisecond exposure to blue light driving depolarization and promoting neural excitation (Boyden et al., 2005). The second generation of ChR2, hChR2(H134R), has an improved protein expression and larger steady state current (Nagel et al., 2005). Recent studies have utilised this variant to directly control vagal afferent modulation *in vivo*. For instance, Williams et al. (2016) activated vagal GLP1R neurons and revealed their function in sensing gastric distension in anaesthetized mice. In 2018, Han et al. (2018) demonstrated distinct roles of right and left NG in food intake in conscious mice by triggering vagal afferent activation, identifying upper gut vagal afferent function in reward circuits and dopamine release via the right NG (Han et al., 2018). In addition, Bai et al. (2019) selectively activated vagal afferent populations expressing *Glp1R* as well as *Oxtr*, and discovered GI vagal afferents subtypes that control feeding (Bai et al., 2019). Besides hChR2(H134R), other improved variants of ChR2 with different kinetics, light wavelength and exposure time are currently available for use (Fenno et al., 2011; Yizhar et al., 2011).

In contrast, halorhodopsins (e.g., NpHR) stimulate neuronal hyperpolarization and inhibit neuronal activity upon yellow/green light exposure (Zhang et al., 2007). Halorhodopsins facilitate anion influx (Cl^–^) into the cells and require constant light exposure to maintain the active state (Zhang et al., 2007). Improved variants of NpHR, such as eNpHR2.0 and eNpHR3.0, exhibit an enhanced membrane localization, increased peak photocurrent, and a high level of gene expression without toxicity compared to the wild type NpHR, in mammalian cells (Gradinaru et al., 2008, 2010). With the opposite function to channelrhodopsins, a combination of ChR2 and NpHR creates a on and off switch to direct neural activity. Kaelberer et al. (2018) demonstrated this scenario using ChR2 to evoke vagal firing, mimicking sucrose-induced vagal activation during signal transduction in the intestine, and eNpHR3.0 to inhibit vagal firing induced by sucrose.

Bacteriorhodopsins (e.g., Arch), in contrast to the other two forms, are proton pumps that enable proton efflux into the extracellular matrix, thus causing hyperpolarization and totally diminishing neural activity (Chow et al., 2010). A major concern with the use of bacteriorhodopsins is the possible extracellular matrix pH alteration due to proton efflux (Chow et al., 2010). Furthermore, a chimeric optogenetic tool, called OptoXR, has been developed by fusing synthetic opsins and GPCRs to facilitate interrogation of biochemical pathways through GPCR Gs and Gq signalling pathways (Airan et al., 2009). To date, no studies have reported the use of these opsins to investigate GI vagal afferent roles.

The use of optogenetics requires a well-designed experimental plan to avoid bias in result interpretation. Variation of protein expression level and activity, side effects of light exposure and tissue heating (Owen et al., 2019), the depth of targeted tissue and instrumental set up can be confounding factors (Fenno et al., 2011). Although optogenetics provides temporal control of neural activity modulation, consideration should be made if long-term stimulation is required. Long term potentiation may cause alteration in neural plasticity and homeostatic adaptation (Allen et al., 2015). Thus, it may alter phenotypes or behavioural responses during observation. The use of slow ChR2 (Schultheis et al., 2011) or chemogenetic can be an alternative if long-term potentiation is required.

Chemogenetics refers to the use of designer receptors activated by designer agonists to modulate neural activity. The majority of chemogenetic tools are based on GPCRs. Designer receptors activated by designer drugs (DREADDs) are the third generation of GPCR-based chemogenetic agents, with downstream effects mediated through classical GPCR (Gq, Gi, or Gs) (Armbruster et al., 2007) or β-arrestin signalling pathways (Allen and Roth, 2011; Nakajima and Wess, 2012). The first DREADD family was created based on human M3 muscarinic receptor (hM1Dq, hM2Di, hM3Dq hM4Di, and hM5Dq) (Armbruster et al., 2007). Gq-DREADDs cause excitatory effects on neuronal activity by mediating calcium (Ca^2+^) influx, while presynaptic inhibitory effects and silencing are obtained by Gi-DREADD activation mediated by cAMP β/γ-GIRK signalling (Armbruster et al., 2007; Roth, 2016). For instance, Bai et al. (2019) selectively activated vagal afferent subtypes in the stomach and small intestine using hM3D-Gq. On the other hand, Gs-DREADDs influence cAMP-signalling to modulate neural activity (Guettier et al., 2009). Han et al. (2018) used hM3D-Gs to simultaneously activate vagal afferents innervating the upper gut in both the NG and their targets in the brainstem.

Muscarinic receptor-based DREADDs are activated by clozapine-N-oxide (CNO) (Armbruster et al., 2007). CNO is an inert chemical actuator with rapid CNS penetration and distribution in mice (Bender et al., 1994). The use of CNO permits a longer time-course to control neural activity, as well as simultaneous activation of cells expressing DREADD in different locations. The ease of CNO administration (e.g., via oral intake) also makes the DREADD-based approach less laborious compared to optogenetics. If a strict temporal control is required, another class of chemogenetic tool based on ligand-gated ion channels that modulate neural activity via ionic conductance can be used (Magnus et al., 2011; Sternson and Roth, 2014). Furthermore, the level of basal activity, receptor desensitisation or downregulation, and side effects due to clozapine back metabolism should be taken into consideration when designing chemogenetic experiments (Roth, 2016).

In addition, a thermal-driven approach termed as thermogenetic is currently under development. Similar to its predecessors, thermogenetics permits controlled activation or inhibition of neuronal activity using thermal stimulus (Bernstein et al., 2012). Molecules with inhibitory effects, e.g., *Drosophila melanogaster* dynamin GTPase (Kitamoto, 2001) and excitatory effects from the thermoTRP family (Dhaka et al., 2006), e.g., *Drosophila melanogaster* TRPA1 (Viswanath et al., 2003; Hamada et al., 2008), rat TRPM8 (Peier et al., 2002; Peabody et al., 2009) and snake TRPA1 (Ermakova et al., 2017), have been discovered. Studies have demonstrated a successful thermal-induced neural activity modulation in drosophila, zebra fish and mouse cultured neurons, but no evidence in *in vivo* models have been reported (Bath et al., 2014; Ermakova et al., 2017).

### Direct Imaging of Vagal Afferent Activity

Communication between neurons occurs via a neurotransmission process. Four classes of genetically encoded indicators have been developed to detect four stages of neurotransmission: pH changes during vesicle fusion (GEPI), neurotransmitter release (GETI), voltage changes (GEVI) and calcium entry (GECI) (Lin and Schnitzer, 2016). These indicators use fluorescence resonance energy transfer (FRET; or single fluorophore-based), of which fluorophore emission will be shifted upon target recognition, allowing visualisation. Recent findings have demonstrated the use of GETI and GECI to decrypt GI vagal afferent signalling. GETI visualises neurotransmitter movement from specific presynaptic to postsynaptic cells and vice versa (Liang et al., 2015). The current available GETIs are glutamate indicators, such as FLIPE, SuperGluSnFR, and iGluSnFR, which exhibit different sensitivity towards glutamate binding (Okumoto et al., 2005; Hires et al., 2008; Marvin et al., 2013). A single fluorophore glutamate sensor, iGluSnFR, has been used to visualise glutamate transmission and identified synaptic connexion between EEC and vagal afferent terminals *in vitro* (Kaelberer et al., 2018). Furthermore, GECIs detect calcium, a second messenger for neurotransmitters and membrane depolarization in neurons, entry during an AP (Grienberger and Konnerth, 2012). GCaMP, a family of single fluorophore GECI, is often used for *in vivo* calcium imaging. For instance, GCaMP3 (Tian et al., 2009) was used to identify vagal GLP1R responses to gastric stretch in a non-intact vagal system in mice (Williams et al., 2016).

### *In vitro* Remodelling

The advancement of stem cell technology has made *in vitro* remodelling at organ level possible. Stem cell-derived three dimensional cell culture or organoids possess some of the key characteristics at the cellular, anatomical or functional level of a real organ (Rossi et al., 2018). The use of organoids to investigate vagal afferent function was recently introduced. Termed as “a gut-brain circuit in a dish”, Kaelberer et al. (2018) simulated the development of synaptic connexions between neuropods and vagal afferents *in vitro* using epithelial intestinal organoids. To date, generation of epithelium-derived gastrointestinal organoids for the oesophagus (DeWard et al., 2014), stomach fundus (McCracken et al., 2017), corpus (Stange et al., 2013; Bartfeld et al., 2015), and pyloric antrum (McCracken et al., 2014; Noguchi et al., 2015), small intestine (Sato et al., 2009; Spence et al., 2011), and colon (Sato et al., 2011) are possible. Epithelial organoids permit a close investigation of synapse formation and the interactions involving vagal afferent terminals, particularly the mucosal endings with the gut epithelial cells. This system provides a tool to examine mechanisms of signalling transduction as may occur in the viscera, such as the interaction with gut hormone secreting cells, for instance.

## Conclusion

Recent advances in technical approaches have made selective targeting and delineating vagal afferent function possible. It is well established that isolating vagal afferent subtypes has been a major challenge that hampers our progress in understanding their function. In recent years, viral-mediated neural tracing, combined with the use of Cre-dependent constructs and Cre-mouse lines, has produced a highly selective approach to target vagal afferent populations. In combination with opto/chemogenetic, calcium imaging and behavioural analysis, we are now able to investigate the role of vagal afferent subtypes *in vivo* and map the neural circuitry. This combination of tools, in a sequence, creates flexible yet excellent experimental frameworks to thoroughly examine vagal afferent function. Although neural tracing is robust, this approach may not be suitable to address all subtypes of vagal afferent due to feasibility of site-specific injection or the presence of sub-subtypes. In this case, the use of transgenic animal models may be an advantage, given the possibility of generating a new line based on the genetic profile of vagal afferent subtypes. It is important to note that the initial step to identify vagal afferent genetic profiles also involves neural tracing to isolate candidate vagal afferent neurons. This shows that integration of different techniques can boost the development of new tools and aid the progress of understanding vagal afferent function.

Despite the current advancement, there is still much to learn about the role of GI vagal afferents in gut function and feeding behaviour. One interesting question is how GI vagal afferent subtypes interact with each other to maintain gut function or respond to food-related signals. While the current studies mainly focus on manipulating a single subtype, incorporation of other site specific recombinase, such as Flp/FRT, Dre/rox and Vika/vox, may allow us to investigate integrative function of different populations in one mouse in the future. In fact, a study has reported a creation of those four systems in one single reporter mouse line (Karimova et al., 2018). Furthermore, the discovery of an optoribogenetic candidate that allows light-driven gene expression control (Weber et al., 2019) opens the opportunity to manipulate endogenous expression of ion channels and receptors. Although speculative, this could aid investigation of the sensing mechanisms of GI vagal afferents and their plasticity towards meal-related stimuli *in vivo*. With these tools, currently in the pipeline, our understanding on GI vagal afferent function could be greatly increased in the near future.

## Author Contributions

YW wrote the review under the supervision of GL and AP. GL and AP edited the manuscript and provided assistance with the structure of the review. All authors contributed to the manuscript and approved the submitted version.

## Conflict of Interest

The authors declare that the research was conducted in the absence of any commercial or financial relationships that could be construed as a potential conflict of interest.

## Footnotes

1 http://www.creportal.org

## Abbreviations

AAV: adeno-associated virus
AgRP: Agouti-related protein
BAC: bacterial artificial chromosome
CAP: capsaicin
CCK: cholecystokinin
CCKR: cholecystokinin receptor
CCK-SAP: cholecystokinin-saporin
ChR2: channelrhodopsin-2
CNO: clozapine-N-oxide
CNS: central nervous system
DMV: dorsal motor nucleus of the vagus
DREADDs: designer receptors activated by designer drugs
dsDNA: double stranded DNA
ECC: enterochromaffin cells
EEC: enteroendocrine cells
ENs: enteric neurons
GECI: genetically encoded calcium indicator
GETI: genetically encoded neurotransmitter release indicator
GI: gastrointestinal
GLP1R: glucagon-like peptide 1 receptor
GPCR: G-protein coupled receptor
GPR65: G-protein receptor 65
HSV: herpes simplex virus
ICC: interstitial cells of Cajal
IGLE: intraganglionic laminar endings
IMA: intramuscular arrays
mRNA: messenger RNA
NG: nodose ganglia
NpHR: *Natromonas pharaonis* halorhodopsin
NTS: nucleus tractus solitarius
PNS: peripheral nervous system
PRV: pseudorabies virus
RABV: rabies virus
RNAi: RNA interference
SDA: subdiaphragmatic vagal deafferentation
SDV: subdiaphragmatic vagotomy
siRNA: small interfering RNA
ssRNA: single stranded RNA
TRPA1: transient receptor potential ankyrin 1
TRPM8: transient receptor potential subfamily M member 8
TRPV1: transient receptor potential vanilloid 1
VAN: vagal afferent neurons
VGLUT: vesicular glutamate transporter.
